# Influenza A H5N1 Detection

**DOI:** 10.3201/eid1108.041317

**Published:** 2005-08

**Authors:** Enders K.O. Ng, Peter K.C. Cheng, Antia Y.Y. Ng, T.L. Hoang, Wilina W.L. Lim

**Affiliations:** * Department of Health, Hong Kong Special Administrative Region, China;; † National Institute of Hygiene and Epidemiology, Hanoi, Vietnam

**Keywords:** human influenza A H5N1 virus, real-time PCR, nested RT-PCR, LightCycler, molecular detection, clinical samples

## Abstract

We developed a sensitive and rapid real-time reverse transcription-polymerase chain reaction (RT-PCR) assay to detect influenza A H5N1 virus in clinical samples. This assay was evaluated with samples from H5N1-infected patients and demonstrated greater sensitivity and faster turnaround time than nested RT-PCR.

The first outbreak of highly pathogenic influenza A H5N1 virus in humans occurred in Hong Kong in 1997 and 6 people with confirmed infection died (1). In February 2003, 2 persons who had traveled to Fujian Province in China were hospitalized in Hong Kong with H5N1 infection (2). Early in 2004, an influenza A H5N1 outbreak emerged in Vietnam and Thailand that caused death in humans and epidemics in the poultry industry (3–5). The recent recurrence of influenza A H5N1 prompted us to highlight the need for a highly sensitive, accurate, and rapid diagnostic test for the infection. Such a test would be important, not only in infection control but also to facilitate early antiviral therapy. Conventional diagnostic tools, cell culture, and serologic testing require from 2 days to 2 weeks for results; thus, they are less useful in making therapeutic and infection control decisions. On the other hand, commercially available rapid antigen tests such as Directigen Flu A+B (Becton Dickinson, Sparks, NJ, USA) or Binax NOW (Binax Inc., Portland, ME, USA) are rapid and simple, but subtyping of viruses is not feasible. Molecular diagnosis of influenza by reverse transcription-polymerase chain reaction (RT-PCR) provides a sensitive and rapid means for detection and has facilitated the typing and subtyping of viruses. Previously, researchers developed tests to detect H5N1 virus by using conventional RT-PCR (6–8) and confirmed the results by Southern blot analysis (6) or restriction fragment length polymorphism–based strategy (8). Although real-time RT-PCR based on the avian H5 gene was developed, the assay has not been evaluated on human clinical specimens (9). We developed a highly sensitive, rapid, and accurate real-time RT-PCR assay to directly diagnose influenza A subtype H5 in human clinical samples. When we evaluated this system using clinical samples from patients infected with H5N1 in Hong Kong and Vietnam, we found it was more sensitive and faster in detecting the virus than the nested RT-PCR that we used previously (3).

## The Study

Our real-time RT-PCR is a multiplex assay that employs a mixture of 2 sets of inhouse designed primers and dual-labeled fluorescent probes that specifically target 2 different regions of the HA gene of H5N1. The primer and probe sets were designed by using the Primer Express software program (Applied Biosystems, Foster City, CA, USA). Both primer and probe sets designs were based on some of the sequences of the recent Vietnam H5N1 strains (e.g., A/Viet Nam/1194/2004). Multiple alignments of previous and recent H5N1 strains were performed to minimize primer mismatch. Multiplex performance was maximized by selecting primers and probes with uniform melting temperatures and minimal cross-hybridization potential. The primer and dual-labeled fluorescent probe sequences are shown in Table 1. This assay is a 2-step, real-time RT-PCR system. Viral RNA is reverse transcribed with random hexamers, followed by real-time PCR. Briefly, viral RNA was extracted from 140 μL clinical specimens in phosphate-buffered saline or viral transport medium with a viral RNA mini kit (Qiagen, Hilden, Germany) and a final elution with 60 μL AVE buffer. Reverse-transcriptase reactions contained 4.2 μL RNA extract, 2 μL 10× PCR buffer (Applied Biosystems), 2.5 μM random hexamer primers (Applied Biosystems), 20 units RNase inhibitor (Applied Biosystems), and 1 μL (50 U) MuLV reverse transcriptase (Applied Biosystems).

**Table 1:** Primers and dual-labeled fluorescent probes of the H5 real-time reverse transcription–polymerase chain reaction assay

Primer/probe	Target	Primer/probe sequence (5´ to 3´)	Nucleotide position*
H5-1F		TGCCGGAATGGTCTTACATAGTG	266–288
H5-1R	HA† gene	TCTTCATAGTCATTGAAATCCCCTG	347–323
H5-1P		f†-AGAAGGCCAATCCAGTCAATGACCTCTGTTA-xp†	290–320
H5-2F		GTGGCGAGCTCCCTAGCA	1615–1632
H5-2R	HA gene	TCTGCATTGTAACGACCCATTG	1695–1674
H5-2P		f-TGGCAATCATGGTAGCTGGTCTATCCTTATGG-xp	1634–1665

*Based on some of the Vietnam H5N1 strains (e.g., A/Viet Nam/1194/2004). †HA, hemagglutinin; f, 6-carboxyfluorescein; xp, carboxytetramethylrhodamine.

Reverse-transcriptase reactions were performed at room temperature for 10 min, then at 42°C for 30 min and at 95°C for 5 min. Five microliters of the cDNA was then used for amplification in the real-time PCR assays. Real-time PCR was carried out with a LightCycler (Roche Diagnostics GmbH, Mannheim, Germany). The real-time PCR reactions (total volume 20 μL) contained 2 μL LightCycler DNA Master Hybridization Probes reaction mix (Roche Diagnostics GmbH), 3 mmol/L magnesium chloride, 250 nmol/L each of the 4 primers, and 125 nmol/L each of the dual-labeled fluorescent probes. The thermal profile used was initiated at 95°C for 10 min (preamplification hot start), followed by 50 cycles of PCR at 95°C for 10 s (denaturation), 56°C for 15 s (annealing), and 72°C for 12 s (extension). At the end of each annealing step, the fluorescent signal of each reaction was measured at a wavelength of 530 nm with the LightCycler fluorimeter. Precautions were taken to prevent cross-contamination of PCR (10).

To test for cross-reactivity, RNA was extracted from isolates or persons with human influenza A H1, H3, H9 subtypes; influenza B; human CoV 229E and OC43; respiratory syncytial virus; rhinoviruses; and enteroviruses. The RNA was then tested by the described real-time RT-PCR. The results showed that the assay was specific for the H5 subtype. The detection sensitivity of the real-time assay was compared with that of 2 other assays by analyzing a serial 10-fold dilution of viral stock with 10^8^ tissue culture infective dose (TCID)_50_/mL of rhabdomyosarcoma cell–culture fluid of a recent H5N1 isolate (A/Viet Nam/1194/2004). The results showed that our real-time RT-PCR assay was the most sensitive of the 3 assays, 10-fold more sensitive than nested RT-PCR (Table 2). We analyzed 18 archived respiratory samples collected ≤10 days after onset of illness from 13 confirmed H5N1–infected patients from Hong Kong in 1997 and 2003 and 10 samples collected on an unknown onset day of illness from 5 confirmed H5N1–infected patients from Vietnam in 2004. All samples were confirmed as H5N1 positive by virus isolation and characterization. Our findings demonstrated that the detection rate of the RT-PCR assay was 100% (28 of 28 samples), whereas that of the nested RT-PCR was 89% (25 of 28 samples). This finding indicates that the real-time assay shows similar or greater sensitivity than the nested RT-PCR. Furthermore, the H5 viral load of the archived clinical samples was determined by a standard curve obtained by plotting from the same series of 10-fold dilutions of virus stock. Viral load analysis showed that the concentration of H5N1 RNA in those clinical specimens ranged from 10^1^ to 10^6^ TCID_50_/mL. Based on the serial sample analysis of 2 patients, the viral load of patient 1 increased 5-fold from day 0 to day 3, whereas the viral load of patient 2 dropped 3-fold from day 4 to day 7 (Figure). Because a limited number of samples were analyzed, the peak of viral load cannot be determined. Studies have reported that the viral load in nasopharyngeal aspirate of the H5N1 patients was lower than in those with H3N2 infection in 2003 (11). To determine whether the viral load of H5N1 patients in 1997 and 2004 is different from that of H3N2 patients, clinical specimens from these 2 groups of patients were analyzed by using a quantitative real-time RT-PCR assay that targeted the M gene of influenza A (12). Similarly, viral load was determined by a standard curve obtained by plotting from series of 10-fold dilutions of a H3N2 virus stock. The mean viral load of 22 patients with H3N2 virus infection was 1.5 × 10^6^ TCID_50_/mL, whereas the mean viral load of H5N1 patients was 1.6 × 10^5^ TCID_50_/mL in both 1997 and 2004. Thus, 10-fold lower viral loads were observed in H5N1 patients in both 1997 and 2004 (*t* test, p<0.05). These data are consistent with previous data that showed rapid diagnostic methods for influenza are less sensitive for H5N1 detection, likely due to lower viral loads.

**Table 2:** Detection sensitivity of polymerase chain reaction and rapid antigen test for H5N1*

Test	Dilution†					
10^3^	10^4^	10^5^	10^6^	10^7^	10^8^
Rapid antigen test‡	+	+	–	–	–	–
Conventional nested RT-PCR	+	+	+	+	–	–
Real-time RT-PCR	+	+	+	+	+	–

*RT-PCR, reverse transcription–polymerase chain reaction. †Serial 10-fold dilution with a concentration of 10^8^ 50% tissue culture infective doses/mL of rhabdomyosarcoma cell culture fluid. ‡Binax NOW; +, positive; –, negative.

**Figure:**
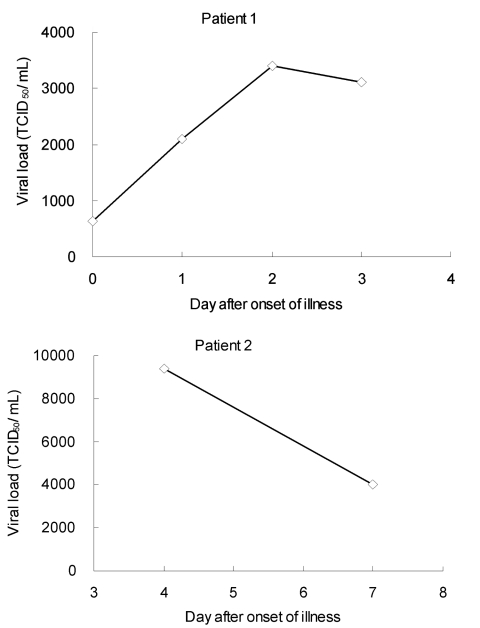
Serial quantitative analysis for influenza A H5N1 virus in respiratory samples from 2 patients with H5N1 in 1997 by quantitative real-time reverse transcription–polymerase chain reaction assay. TCID_50_, 50% tissue culture infective dose.

## Conclusions

In this study, we demonstrated that our new multiplex real-time RT-PCR assay that specifically targets 2 different regions of the H5 gene is more sensitive than nested RT-PCR and even more sensitive than real-time RT-PCR with a single set of primers and probes (unpub. data). Our data prove that the assay is specific for H5 subtype and capable of detecting and quantifying H5 RNA in clinical samples from patients obtained during different outbreaks (1997, 2003, and 2004). Unlike nested RT-PCR, the real-time assay not only reduces the risk for contamination but also reduces turnaround time to 1–2 hours, 3 times faster than the nested RT-PCR. In conclusion, our study demonstrates that our real-time RT-PCR assay is rapid, specific, and relatively sensitive for directly detecting influenza A subtype H5 virus and may be useful in routine diagnostic testing.
